# Isoliquiritigenin Inhibits Atherosclerosis by Blocking TRPC5 Channel Expression

**DOI:** 10.1155/2020/1926249

**Published:** 2020-04-07

**Authors:** Jie Qi, Jianguo Cui, Baobin Mi, Xiaohong Yan, Wenwen Xu, Hui Ma, Qingtan Zhang, Fang Xu

**Affiliations:** ^1^Department of Geriatrics, Binzhou Medical University Hospital, No. #661 Yellow River Road No. 2, Binzhou, Shandong 256603, China; ^2^Department of Pathophysiology, Binzhou Medical University, 346# Guanhai Road, Yantai, Shandong 264003, China

## Abstract

Isoliquiritigenin (ISL) is a flavonoid isolated mainly from the licorice plant, a traditional Chinese herb. ISL has shown anticancer, anti-inflammatory, antioxidant, and antidiabetic activities. However, the pharmaceutical effects of ISL on atherosclerosis are seldom explored. In this study, we used apolipoprotein *E* (ApoE) knockout mouse model and angiotensin II- (Ang II-) stimulated vascular smooth muscle cells (VSMCs) to elucidate the pharmacological mechanism of ISL to inhibit atherosclerosis. We found that in ApoE^−/−^ mice ISL could attenuate atherosclerotic lesion, reduce serum lipid levels, and inhibit TRPC5 expression. In vitro, ISL inhibited Ang II-stimulated proliferation of VSMCs and suppressed Ang II-induced TRPC5 and PCNA expressions in a dose-dependent fashion. In conclusion, our findings provide novel insight into the pharmacological effects of ISL on atherosclerosis and suggest that ISL is beneficial for cardiovascular protection.

## 1. Introduction

Isoliquiritigenin (ISL) is a flavonoid compound from *Glycyrrhiza glabra*, the licorice plant of the traditional Chinese herb [1]. Studies have shown that isoliquiritigenin has a variety of activities such as anticancer [2], antibacterial [3], antivirus [4], antiasthma [5], anti-inflammatory [6], antidiabetic [7], and antioxidant activities [8]. Furthermore, ISL could reduce low-density lipid (LDL) via antioxidant activity [9]. In addition, ISL has been shown to inhibit the proliferation and induce the apoptosis of tumor cells [10–13].

Vascular smooth muscle cells (VSMCs) play an important role to maintain vascular tension and basic physiological functions in tunica media. In the development of atherosclerosis, VSMCs can transform to dedifferentiated phenotype after intimae damage and have increased the ability of proliferation and migration [14]. The phenotype change of VSMCs participates in the process of plaque forming and atherosclerosis acceleration [15]. However, whether ISL could suppress the proliferation of VSMCs remains obscure.

As an important second messenger, Ca^2+^ is controlled by different types of Ca^2+^ channels and transporters in the membrane of VSMCs. Among them, store-operated channels (SOCs) can activate the transcription of early response genes and affect the proliferation, migration, and synthesis of excessive extracellular matrix [16]. Transient receptor potential canonical 5 (TRPC5) channel is a representative of SOCs mainly localized in VSMCs and is activated by IP_3_ (inositol 1,4,5-trisphosphate) to induce slow and continuous calcium influx [17]. Interestingly, a recent study reported that ISL induced vasodilation by activating Ca^2+^-activated K^+^ channels in VSMCs [18]. Therefore, we hypothesized that ISL may modulate the TRPC5 channel to regulate the proliferation of VSMCs. In this study, we used the apolipoprotein *E* (ApoE) model and angiotensin II- (Ang II-) induced VSMCs model to elucidate the pharmacological mechanism of ISL to inhibit atherosclerosis.

## 2. Materials and Methods

### 2.1. Animal Model

All animal experiments were approved by the Animal Care and Use Committee of Binzhou Medical University. C57BL/6J mice and ApoE knockout C57BL mice (male, 6-week-old) were purchased from the Laboratory Animal Center of Peking University (Beijing, China). The mice were bred and maintained in barrier facilities at 24°C–26°C with 12 h light/12 h dark cycle. All mice were treated following the Chinese Institutes of Health Guide for the Care and Use of Laboratory Animals. ApoE^−/−^ mice were randomly divided into 2 groups (*n* = 20): model group and ISL group, and fed with a regular chow diet (21% fat + 0.15% cholesterol). ISL group mice were lavaged with ISL (20 mg/Kg/d). C57BL/6J mice were chosen to be the control group (*n* = 20), which were fed ordinary food. All the mice were killed by euthanasia after 12 weeks.

### 2.2. Reagents

ISL (MB2209, Dalian, China) was purchased from Melone and dissolved in dimethyl sulfoxide (DMSO) to make a stock solution of 0.25 mol/l. Then, it was diluted to the final concentrations in a culture medium, and DMSO final concentration was <0.1% (v/v) to avoid its toxic effect on the growth of cells. Primary antibodies for TRPC5 and MOMA-2 were from Abcam, *α*-SMA antibody was from Bioss (China), proliferating cell nuclear antigen (PCNA) antibody was from Affinity, and GAPDH antibody was from Epitomics.

### 2.3. Blood Lipid Analysis

The retro-orbital plexus method was used to obtain blood samples. Serum levels of total cholesterol (TC), triglyceride (TG), high-density lipoprotein cholesterol (HDL-C), and low-density lipoprotein cholesterol (LDL-C) were measured by using commercial enzymatic methods with the kits on RX-30 device (Nihon Denshi, Tokyo, Japan).

### 2.4. Histology and Morphometric Analyses

The aortic roots were collected and stored at −80°C. Samples were sliced into 25 sections (10 *μ*m thick). The sections were stained with hematoxylin-eosin, and plaque area (PA), luminal area (LA), and the percentage of corrected plaque area (PA/LA) were measured using Image-Pro Plus software. In addition, the sections were subjected to immunohistochemical (IHC) staining with TRPC5 and integrated option density (IOD) of TRPC5 staining was measured using Image-Pro Plus software.

### 2.5. Cell Culture

VSMCs were isolated from C57BL/6J mice thoracic aorta as described (Rodriguez et al., 2007). Cells were cultured in Dulbecco's modified Eagle's medium (DMEM)/high glucose (HyClone, USA) supplemented with 20% fetal bovine serum (FBS, Gibco, USA) in a humid atmosphere with 5% CO_2_ at 37°C. VSMCs were characterized by *α*-actin immunocytochemistry assay (Bioss, Beijing, China). Cells from generations 4–9 were used for the experiments. VSMCs were divided into 5 groups: control group (treated with 2% FBS), Ang II 10^−6^ mol/l group (treated with angiotensin II at 10^−6^ mol/l), Ang II 10^−6^ mol/*l* + 10 *μ*mol/l ISL group, Ang II 10^−6^ mol/*l* + 20 *μ*mol/l ISL group, Ang II 10^−6^ mol/*l* + 40 *μ*mol/l ISL group, and Ang II 10^−6^ mol/*l* + 60 *μ*mol/l ISL group.

### 2.6. Cell Proliferation Assay

VSMCs were seeded at 2,000 cells/well in 96-well plates overnight. After the treatment, cells were incubated with 10 *µ*l/ml CCK8 for 4 h, and then the absorbance at 450 nm was measured using a reader.

### 2.7. PCR

Total RNA was extracted from aorta using Trizol. Real-time PCR was performed using SYBR Green on a Rotor-Gene 3000 Run StepOnePlus^™^ Real-Time PCR System (Corbett, Australia). The sequences of primers were as follows: TRPC5, forward 5′-ACAAAAAGGTCAACTACTCACCG-3′, reverse 5′-CAGTGGCATAGTCCCCCTTCT-3′; GAPDH, forward 5′-AACTGCTTAGCACCCCTGGC-3′, reverse 5′-ATGACCTTGCCCACACAGCCTT-3′. Quantitative measurements were determined using the ΔΔCt method and GAPDH was used as an internal control.

### 2.8. Western Blot Analysis

Proteins were extracted and 40 *μ*g from lysates per lane was loaded on a 10% SDS-polyacrylamide electrophoresis gel and then electrophoresed and transferred onto polyvinylidene fluoride (PVDF) membranes. The membranes were blotted with specific antibodies against TRPC5, PCNA, and GAPDH at 4°C overnight and then incubated with horseradish peroxidase-conjugated secondary antibody for 1 h. Immunoreactivity was detected by using the enhanced chemiluminescence (ECL) method. Protein content was calculated by densitometry using LabWorks software.

### 2.9. Statistical Analysis

All data are presented as mean ± SD and analyzed by SPSS 13.0 software. For comparison among groups, one-way ANOVA was applied, followed by LSD test. *P* < 0.05 was regarded as significant.

## 3. Results

### 3.1. ISL Improved the Health Condition in ApoE^−/−^ Mice

**Figure 1:**
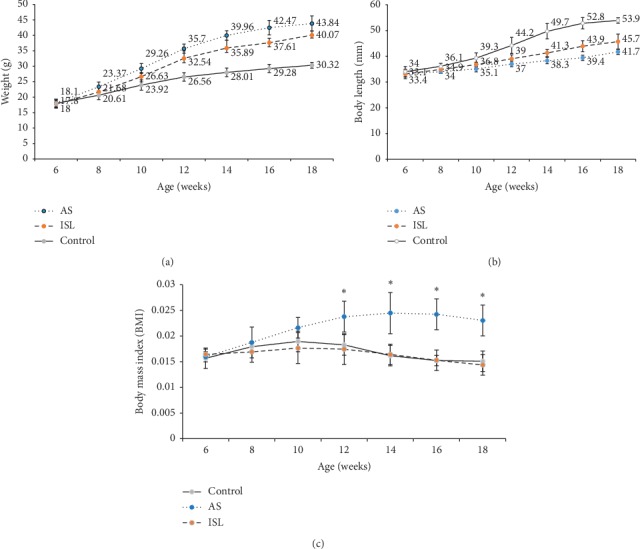
Body weight and length and body mass index of mice in three groups (*n* = 20). (a) Body weight in different groups of mice of different ages. (b) Body length in different groups of mice of different ages. (c) Body mass index (BMI) in different groups of mice of different ages. Control: control group; AS: atherosclerosis group; ISL: isoliquiritigenin group. ^*∗*^*P* < 0.05 compared to AS at the age of 12, 14, 16, and 18 weeks.

The general condition of the control group was the best in all mice. ApoE^−/−^ mice in the AS model group showed the lowest growth and the weight increased faster (Figures 1(a) and 1(b)). Compared to the AS model group, ApoE^−/−^ mice in the ISL group had better body mass index (BMI) at 12–18 weeks (Figure 1(c), *P* < 0.05).

### 3.2. ISL Improved Blood Lipids and Atherosclerosis Lesion in ApoE^−/−^ Mice

**Figure 2:**
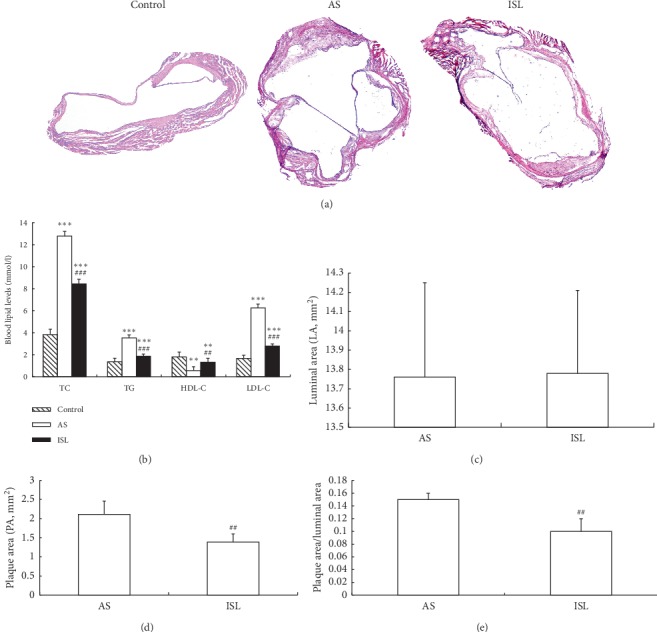
Histology and morphometric analyses of atherosclerotic lesion (*n* = 20). (a) HE staining of aortic tissues (×40). No obvious aortic lesion was observed in control group. AS group showed unstable plaques which were diffusing all the lumen of aortas, but they were improved in ISL group. (b) The levels of blood lipids (unit as mM) in different groups. (c) Comparison of luminal area (LA, unit as mm^2^) in AS and ISL groups. (d) Comparison of plaque area (PA, unit as mm^2^) in AS and ISL groups. (e). Comparison of the ratio of PA/LA in AS and ISL groups. ^*∗∗*^*P* < 0.01, ^*∗∗∗*^*P* < 0.001, compared to control group; ^##^*P* < 0.01, compared to AS group. Control: control group; AS: atherosclerosis group; ISL: isoliquiritigenin group.

HE staining indicated that the atherosclerosis model group had unstable plaques which were diffusing all the lumen of aortas, but they were improved in the ISL group (Figure 2(a)). Serum levels of TC, TG, and LDL-C were the highest while HDL-C level was the lowest in atherosclerosis model group. TC, TG, and LDL-C levels significantly declined and the HDL-C level increased in the ISL group compared with the model group (Figure 2(b)). The aortic intima of the model group was thickened, and plaque formation, significant luminal stenosis, more lipid pools, and meager fibrous caps within plaques were observed in the model group. In contrast, these lesions were slight, plaque areas were small, lipid pools were thin, and fewer foam cells and inflammatory cells were found in the ISL group, and plaque area was reduced (Figures 2(c)–2(e)).

### 3.3. ISL Inhibited TRPC5 Expression in Atherosclerosis Model Mice

**Figure 3:**
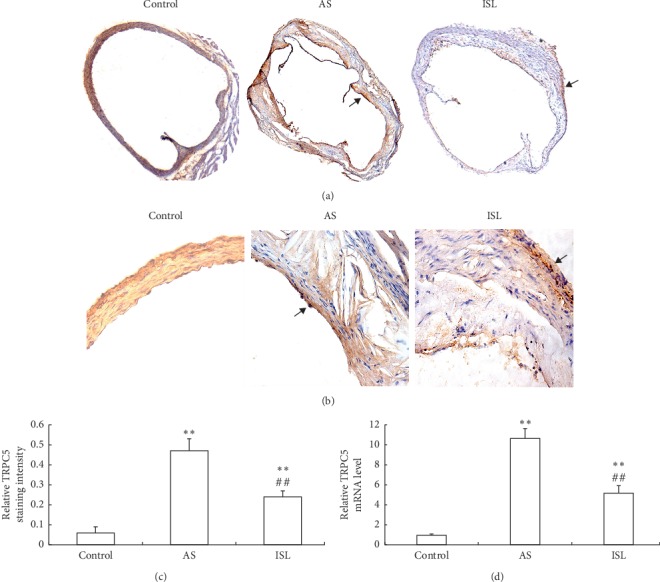
TRPC5 expression in mice of three groups. (a) TRPC5 staining in aortas of each group (×40). Staining area was indicated by the arrows. (b) TRPC5 staining in aortas of each group (×400). Staining area was indicated by the arrows. (c) Comparison of TRPC5 staining intensity in different groups. (d) Comparison of TRPC5 mRNA levels in different groups. ^*∗∗*^*P* < 0.01, compared to control group; ^##^*P* < 0.01, compared to AS group. Control: control group; AS: atherosclerosis group; ISL: isoliquiritigenin group.

Immunohistochemical analysis showed that TRPC5 was located both in tunica media and in VSMCs which migrated into artery intima (Figures 3(a) and 3(b)). TRPC5 staining was significantly stronger in the model group compared to the control group but was significantly weaker in the ISL group compared to the model group (Figure 3(c)). PCR analysis confirmed that the relative TRPC5 mRNA level was significantly higher in the model group compared to the control group but was significantly lower in the ISL group compared to the model group (Figure 3(d)).

### 3.4. ISL Inhibited Ang II-Induced Proliferation of VSMCs

**Figure 4:**
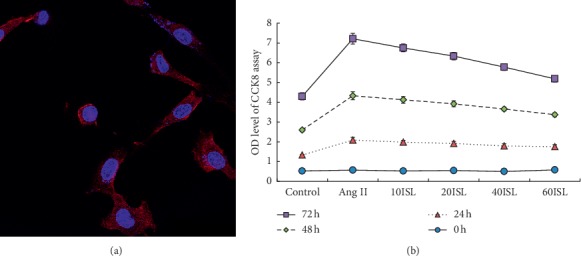
ISL inhibited the proliferation of VSMCs. (a) Identification of VSMCs isolated from C57BL/6J mice aortas (staining ×400). (b) CCK8 assay of the proliferation of VSMCs. Values are expressed as means ± SD (*n* = 5). Control: control group; Ang II: Ang II group; 10ISL: Ang II 10^−6^ mol/*l* + 10 *μ*mol/l ISL group; 20ISL: Ang II 10^−6^ mol/*l* + 20 *μ*mol/l ISL group; 40ISL: Ang II 10^−6^ mol/*l* + 40 *μ*mol/l ISL group; 60ISL: Ang II 10^−6^ mol/*l* + 60 *μ*mol/l ISL group.

We isolated VSMCs from C57BL/6J mice aorta and immunocytochemistry confirmed the identification of VSMCs (Figure 4(a)). CCK8 assay showed that VSMCs proliferation was activated by angiotensin II. However, Ang II-stimulated VSMCs proliferation was inhibited by ISL in a concentration-dependent manner (Figure 4(b)).

### 3.5. ISL Inhibited TRPC5 and PCNA Expression in Ang II-Stimulated VSMCs

**Figure 5:**
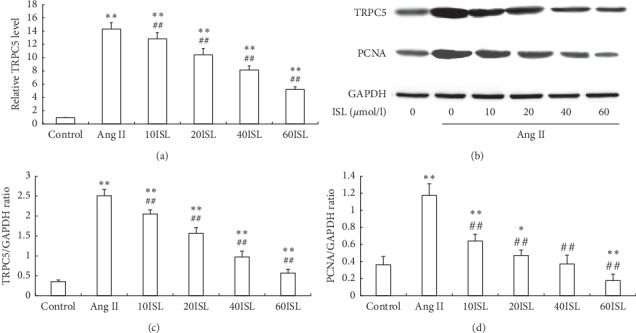
ISL inhibited TRPC5 and PCNA expressions in VSMCs. (a) The mRNA levels of TRPC5 in different groups. (b) The protein levels of TRPC5 and PCNA were determined by Western blot analysis. GAPDH was loading control. The representative images were shown from 5 independently performed tests. (c) Densitometry analysis of TRPC5 protein expression. (d) Densitometry analysis of PCNA protein expression. Values are expressed as means ± SD (*n* = 5). ^*∗∗*^*P* < 0.01, compared to control group; ^##^*P* < 0.01, compared to Ang II group. Control: control group; Ang II: Ang II group; 10ISL: Ang II 10^−6^ mol/*l* + 10 *μ*mol/l ISL group; 20ISL: Ang II 10^−6^ mol/*l* + 20 *μ*mol/l ISL group; 40ISL: Ang II 10^−6^ mol/*l* + 40 *μ*mol/l ISL group; 60ISL: Ang II 10^−6^ mol/*l* + 60 *μ*mol/l ISL group.

Real-time PCR showed that Ang II significantly induced the expression of TRPC5 mRNA in VSMCs, but ISL reduced Ang II-stimulated TRPC5 mRNA expression (Figure 5(a)). Western blot analysis showed that Ang II significantly induced the expression of TRPC5 protein in VSMCs, but ISL reduced Ang II-stimulated TRPC5 protein expression (Figures 5(b) and 5(c)). In addition, Ang II significantly induced the expression of PCNA protein in VSMCs, but ISL reduced Ang II-stimulated PCNA protein expression (Figure 5(d)).

## 4. Discussion

*Glycyrrhiza glabra* (Licorice) is a traditional medicinal herb widely used with many pharmaceutical effects [19]. ISL is a flavonoid compound isolated from this plant and has a variety of activities [20]. However, the pharmaceutic effects of ISL on atherosclerosis have not been explored. Therefore, in this study, we chose ApoE knockout mice as an atherosclerosis model to investigate the anti-atherosclerosis effects of ISL.

In the present study, we found that ISL could regulate blood lipids of ApoE^−/−^ mice. A previous study showed that licorice flavonoid oil could regulate the expression of lipid metabolism-related genes to ameliorate hyperlipidemia of C57BL/6J mice [21]. Our results showed that ISL significantly improved the weight and blood lipids in ApoE^−/−^ mice fed with a high-fat diet. Furthermore, ISL could inhibit TRPC5 expression not only in high-fat diet-induced atherosclerosis model but also in primary VSMCs stimulated by angiotensin II.

It is known that phenotype changes in VSMCs promote the formation of atherosclerosis [22, 23]. Proliferation and migration of VSMCs are critical for plaque formation and atherosclerosis development. TRPC5 is the main subtype of store-operated channels in the aorta and can be activated by inositol 1,4,5-triphosphate (IP3), leading to continuous calcium influx [17]. TRPC5 could regulate the function of VSMCs [24, 25]. Oxidized low-density lipoprotein (ox-LDL), a risk factor accelerating atherosclerosis, was found to promote VSMCs proliferation and migration, and TRPC5 channels were sensitive to antioxidant [26]. In ApoE^−/−^ mice, blood lipid deposits on the aorta intima increased ox-LDL to stimulate TRPC5 overexpression. In vitro, angiotensin II induced VSMCs proliferation, and internal Ca^2+^ could stimulate TRPC5 channels directly [27]. In our present study, we found that ISL significantly downregulated TRPC5 and PCNA expression in a dose-dependent manner. Thus, we speculate that ISL may inhibit VSMCs proliferation by downregulating TRPC5. However, our study has limitations in that we did not further investigate the signaling pathways which may be responsible for mediating the inhibitory effects of ISL on TRPC5 expression.

Interestingly, it was reported that ISL could inhibit NF-*κ*B and mitogen-activated protein kinases (MAPK) signal pathways [28]. It has been confirmed that the activation of NF-*κ*B and MAPK pathways accelerates the atherosclerosis process and promotes VSMCs proliferation and migration [29]. Oxidative stress and calcium influx may also affect TRPC5 expression. Therefore, further studies are needed to reveal the mechanisms by which ISL modulates TRPC5 expression and inhibits atherosclerosis.

In conclusion, we demonstrate that ISL inhibits TRPC5 overexpression not only in a high-fat diet-induced atherosclerosis mouse model but also in primary VSMCs stimulated by angiotensin II. Furthermore, ISL improved atherosclerosis in the mouse model and inhibited the proliferation of primary VSMCs. These findings provide novel insight into the pharmacological effects of ISL on atherosclerosis and suggest that ISL is beneficial for cardiovascular protection.

## Data Availability

All data are available upon request from the corresponding authors.
